# Two months follow-up of patients with non-critical COVID-19 in Cape Town, South Africa

**DOI:** 10.4102/safp.v64i1.5429

**Published:** 2022-02-10

**Authors:** Andrea S. Mendelsohn, Nikhil Nath, Angela De Sá, Klaus B. von Pressentin

**Affiliations:** 1 Cape Metro Health Services, Retreat Community Health Centre, Western Cape Department of Health, Cape Town, South Africa; 2 Department of Epidemiology and Biostatistics, School of Public Health, Tufts University School of Medicine, Boston, United States of America; 3 School of Public Health and Family Medicine, Faculty of Health Sciences, University of Cape Town, Cape Town, South Africa

**Keywords:** long COVID, primary health care, rehabilitation, mild COVID-19, South Africa

## Abstract

**Background:**

Approximately 10% of coronavirus disease 2019 (COVID-19) patients will experience long COVID. There is no study of long COVID in mild COVID-19 patients in South Africa. This study aimed, firstly, to describe the prevalence of long COVID in mild COVID-19 patients in Cape Town, and, secondly, to document the impact of COVID-19 on patients’ well-being, work, and their access to long COVID treatment.

**Methods:**

In this retrospective cross-sectional study, a random sample of adults diagnosed with mild COVID-19 were called two months post-diagnosis. The participants telephonically completed a standardised survey describing their long COVID symptoms, missed workdays, and health-seeking behaviour. Medical records were reviewed for comorbidities, original COVID-19 symptoms, and treatment.

**Results:**

It was found that 60% of patients with mild COVID-19 had ≥ 1 long COVID symptom, while 35% had ≥ 3 ongoing symptoms for two months. Dyspnoea and fatigue were the most common symptoms. The findings revealed that 52% of employed patients missed work and 25% of patients self-reported non-recovery from their COVID-19. Moreover, 24% of patients consulted a clinician for long COVID, but only 7% of patients received long COVID care in the public sector. Of the 17% of patients requiring additional help for long COVID, 56% were interested in assistance by text message or telephonic consultation.

**Conclusion:**

Over a half of mild COVID-19 patients experienced at least one long COVID symptom for two months and nearly 20% needed additional medical treatment. Very few patients utilised the public sector for long COVID treatment. There is a great need for long COVID treatment in public healthcare services and patients are receptive to remote care.

## Introduction

Long coronavirus disease (COVID) is defined as coronavirus disease 2019 (COVID-19) related symptoms that persist more than 28 days from the onset of acute infection.^1^ Observational studies have reported a wide range in the prevalence of long COVID, between 32.6% and 87.4% of patients reporting at least one persistent symptom between two and four months post-hospitalisation for COVID-19.^2,3,4,5,6^ In the United Kingdom (UK), Sweden and United States (US), a COVID Symptoms Study App was used to follow 4182 people with confirmed severe acute respiratory syndrome coronavirus 2 (SARS-CoV-2) infection, of whom only 13.9% of participants had required hospitalisation.^7^ In this COVID-19 cohort, 13% of those confirmed cases reported symptoms > 28 days, 4.5% had symptoms > 8 weeks, and 2.3% had persistent symptoms > 12 weeks.^7^ In the only South African study to date, the National Institute for Communicable Diseases (NICD) interviewed 1448 adults with confirmed SARS-CoV-2 infection 1-month post-hospital discharge.^8^ In this study, 82.2% of patients reported at least one symptom, with 32.8% having ≥ 4 symptoms 1-month post-discharge.^8^

Overall, it is estimated that 10% of people with acute COVID-19 will continue to have long COVID symptoms.^9^ In the COVID Symptoms Study app analysis, people with ≥ 5 symptoms during the first week of illness were four times more likely to develop long COVID.^7^ Older age > 50 years, the severity of initial disease, female gender, and obesity were also associated with a higher risk of long COVID.^7^ Similarly, the NICD survey reported that pre-existing obesity was a risk factor for self-reported non-recovery, while these patients with ≥ 4 symptoms during the acute illness were five times more likely to have long COVID symptoms in comparison to those with asymptomatic COVID-19.^8^ Black Africans were half as likely to self-report non-recovery at 1-month post-hospitalisation in comparison to white patients.^8^

Globally, long COVID is associated with a range of fluctuating symptoms. Common physical symptoms include cough, fatigue, dyspnoea, myalgia, headaches, anosmia, sore throat, and chest pain.^2,3,7,9,10^ However, diarrhoea, palpitations, skin changes, tinnitus, and neurological changes have also been reported. Psychological and cognitive problems are also common, with multiple studies highlighting an increase in anxiety, depression, post-traumatic stress disorder, memory impairment, and sleep disturbances.^10,11,12,13^ In South Africa, the most common symptoms reported 1-month post-discharge were fatigue (69.8%), shortness of breath (32.0%), headache (17.5%), weakness in arms or legs (17.5%), and poor concentration or confusion (16.0%).^8^

Although Cape Town in South Africa was severely affected by the COVID-19 epidemic – with over 300 000 cases in the Cape metro by September 2021^14^ – community clinicians in the public sector have reported that only a relatively few patients were returning to primary healthcare facilities for treatment of long COVID. This situation is in contrast to the literature and anecdotal reports of higher rates of long COVID in the private sector. It is unlikely that there is a biological difference between South African public sector patients post-COVID-19 and their counterparts in the global north. Several explanations might account for the perceived differences in healthcare utilisation in the South African public sector. However, aside from the NICD study of severe COVID-19 patients post-hospitalisation, there is no data on the prevalence of long COVID in non-hospitalised patients in South Africa.^8^ Leading experts on infectious diseases recommended further research, especially in the South African context with its high burden of comorbid infectious and non-communicable diseases, which may complicate the manifestations of long COVID.^1^

The primary objective of this study is to describe the prevalence of long COVID symptoms in patients with confirmed non-critical SARS-CoV-2 infection in Cape Town, South Africa two months after diagnosis. The second objective is to document the impact of long COVID on patients’ self-perceived well-being and the impact of long COVID on their employment. The third objective is to assess whether public sector patients received medical care for long COVID. If the patients did not receive care, this study aims to find out some of the barriers to the treatment for long COVID in the public sector.

## Methods

Retreat Community Health Centre (CHC) is a 24-h healthcare facility that provides both acute and chronic outpatient primary healthcare. Patients requiring admission are referred to the district hospital. Retreat CHC drains patients from low-income communities and squatter camps in Cape Town’s Southern peninsula and part of the Cape Flats. In total, 653 adults were diagnosed with COVID-19 by polymerase chain reaction (PCR) test at Retreat CHC between 15 December 2020 and 31 January 2021, during the second wave of COVID-19 in South Africa which was dominated by the Beta variant. During that time, public sector PCR testing was limited to all adults ≥ 45 years old, patients of any age with high-risk comorbidities, healthcare workers, and others who work in congregate settings.^15^ Contact tracers called each confirmed SARS-C0V-2 positive patient to identify the household contacts for quarantine; they kept logbooks for recording the contact information of the patients.

For this retrospective cross-sectional study, contact tracers phoned a random sample of adults ≥ 18 years old diagnosed with SARS-CoV-2 infection using PCR test at Retreat CHC 2 months post-diagnosis. Two months were selected to compare the results to similar studies in the literature that also followed up patients after 60 days.^2,3,4^ All adults with confirmed SARS-CoV-2 infection diagnosed at Retreat CHC in the second wave of COVID-19 were eligible to participate. However, contact tracers only called a random sample of the patients they originally notified by telephone between 15 December 2020 and 31 January 2021, as recorded in case logbooks. The contact tracers called 352/653 eligible patients between 15 February 2021 and 31 March 2021. Three attempts were made to contact each patient. Of those patients who were called, 175 patients were reached and they were invited to participate in the study. One patient refused to take part in the study. The remaining 174 patients consented to participate in the study.

The contact tracers telephonically completed a standardised questionnaire with each participant. The patients were asked to rate their general health – if they felt better, worse, or the same as before COVID-19. They were asked about the persistence and severity of common long COVID symptoms including dyspnoea, fever, fatigue, headache, chest pain, gastrointestinal complaints, myalgia, arthralgia, anosmia, ageusia, palpitations, and skin changes. The Modified Medical Research Council (MMRC) Dyspnoea scale was used to rate dyspnoea.^16^ For patients complaining of fatigue, the WHO performance status classification scale was used to grade the participants’ level of activity post-COVID-19.^17^ Work status and the number of workdays missed post-isolation were documented. The patients were asked if they consulted a clinician for persistent COVID-19 symptoms beyond the isolation period. If they did not, they were asked if it was because they felt well or because of any barrier to access healthcare – they believed the CHC would turn away non-emergencies, the wait was too long, or an alternative reason. Finally, the patients were asked if they would like to receive medical help for ongoing symptoms and what type of help they preferred – a traditional clinic visit, a text message interaction, a telephone call, or a community group visit.

Following the survey, we attempted to review the medical folders for all the patients who were surveyed. We managed to locate 157/174 folders. Data extracted from the individual patient medical folders included patient comorbidities, acute COVID-19 symptoms and care, and the number of medical visits at the CHC for persistent COVID-19 symptoms post isolation.

Descriptive statistics were generated for patient demographics, comorbidities, employment status, acute COVID-19 symptoms, number of medical consultations for persistent symptoms post isolation, number of missed workdays, self-reported non-recovery, long COVID symptoms, and the desire for additional medical consultation for long COVID. Multivariate Logistic Regression was used to analyse if individual categorical patient characteristics – gender, age, number of COVID symptoms, initial severity of COVID – were predictive of long COVID symptoms. Odds ratios (ORs) were generated with 95% confidence intervals (CI) (*p* < 0.05). Quantitative data were analysed using STATA (Version 17, College Station, Texas, US).

### Ethical considerations

Ethical approval was obtained from the University of Cape Town’s Faculty of Health Sciences Research Ethics Committee (HREC reference number 861/2020) and the Western Cape Department of Health Provincial Research Committee (reference WC_202102_017).

## Results

We surveyed 174/653 (27%) of PCR-confirmed SARS-CoV-2 infections diagnosed in the second wave of COVID-19 at Retreat CHC 2 months post-diagnosis. There was a mean of 62.3 (6.9) days between diagnosis and survey. Participant baseline demographics and medical comorbidities are described in Table 1. The mean age of participants was 50.3 (13.6) years; 62% were female. Because of Retreat CHC’s patient demographic, 94% of participants self-identified as ‘Coloured’ and 62% were employed. 42% of participants had no comorbidities. Of those with comorbidities, hypertension (48%) and diabetes (22%) were the most common.

**TABLE 1:** Baseline patient characteristics.

Patient characteristics	*n*	%
**Sex**		
Female	108	62.1
Male	66	37.9
**Race**		
Coloured	164	94.3
African	6	3.5
White	1	0.6
Other	3	1.7
**Employment status**		
Employed	107	61.5
Unemployed†	67	38.5
**Co-morbidities** ‡		
None	66	41.5
Diabetes	35	22.0
Hypertension	77	48.4
Ischemic heart disease	7	4.4
COPD/asthma	10	6.3
Prior TB	1	0.6
HIV	1	0.6
Other	7	4.4

Note: Mean age: 50.3; s.d. = 13.6.

s.d., standard deviation; COPD, chronic obstructive pulmonary disease; TB, tuberculosis; HIV, human immunodeficiency virus.

† , Includes pensioner;

‡ , Extracted from 157 folders reviewed.

Nearly all participants – 154/157 (98%) – had mild COVID-19 with outpatient management. The mean oxygenation saturation level at the time of diagnosis was 97% (2.3), with only eight patients having saturations below 93% at the initial time of consultation. See Table 2 for a description of participants’ COVID-19 symptoms and management at the time of illness. The results showed that 42/174 (24%) of participants returned to a clinician post isolation for the management of persistent COVID-19 symptoms. However, only 12/42 (29%) obtained that care at the public CHC where the diagnosis was made. The other 30 participants consulted a private general practitioner for ongoing COVID-19 symptoms. Of those who did not consult a clinician post isolation, 98/132 (74%) participants reported they ‘felt well’. An additional 26/132 (20%) patients believed their symptoms were ‘not serious enough’ to warrant medical treatment. Only two people cited ‘long queues’ and ‘I thought the clinic would turn away non-emergencies’ as a reason for not seeking healthcare. Three participants stated that they were too anxious to return to the CHC again during the COVID-19 epidemic. With respect to the impact of long COVID on work, 56/107 (52%) employed participants missed work because of persistent COVID-19 symptoms in the 2 months following their diagnosis, with the median number of workdays missed beyond the isolation period being 2 days (inter quartile range [IQR]: 5).

**TABLE 2:** COVID-19 episode characteristics.

COVID-19 characteristics	*n*	%
**COVID-19 treatment**		
Outpatient	154	98.1
Hospitalised	3	1.9
**COVID-19 symptoms**		
Cough	112	70.4
Fever	67	42.1
SOB	57	35.9
Myalgia	42	26.4
Headache	52	32.7
Sore throat	65	40.9
Diarrhoea	13	8.2
Loss taste/smell	50	31.5
Vomiting	3	1.9
**Medical consultations for persistent COVID-19 symptoms (post-isolation)**		
0	132	75.9
1	32	18.4
2+	10	5.7
**Missed work because of COVID-19 (excluding isolation)**		
0 days	51	29.3
1+ days	56	32.2

Note: Mean oxygen saturation at diagnosis: 97; s.d. = 2.3 Median days work missed 2 days ; IQR = 5.

s.d., standard deviation; IQR, interquartile range; SOB, shortness of breath.

Two months post diagnosis, 105/174 (60%) participants had at least one long COVID symptom, with 62/174 (35%) reporting three or more ongoing symptoms (Table 3). The most common long COVID symptoms were fatigue (35%), dyspnoea (20%), ageusia (20%), and anosmia (18%) (Table 3). Of participants with fatigue, the mean self-reported level of activity post-COVID-19 on the WHO performance status classification scale was grade 1 (0.7) (grade 1 = restricted in physically strenuous activity but ambulatory and able to carry out work of a light or sedentary nature). The mean level of self-reported dyspnoea was grade 1 (1.0) on the MMRC Dyspnoea scale. Of patients who experienced ageusia and anosmia, 2/34 (6%) participants had complete ageusia and 1/32 (3%) had complete anosmia at two months. Just over 40% complained of partial ageusia (14/34, 41%) and anosmia (14/32, 44%), respectively, at two months, while 12/34 (35%) had ageusia and 11/32 (34.4%) had anosmia for ≥ one month that had resolved by two months.

**TABLE 3:** Long COVID characteristics.

Long COVID characteristics	*n*	%
**General health** †		
Worse than before COVID-19	44	25.6
Same as before COVID-19	108	62.8
Better than before COVID-19	20	11.6
**Long COVID symptoms**		
Fatigue	60	34.5
Dyspnoea	35	20.1
Loss of taste	34	19.5
Loss of smell	32	18.4
Headache	27	15.5
Body aches	26	14.9
Chest pain	19	10.9
Gastrointestinal complaints	21	12.1
Palpitations	18	10.3
Rash	11	6.3
**Number of long COVID symptoms**		
0	69	39.7
1	21	12.1
2	22	12.6
3+	62	35.6
**Do you want additional medical help for persistent COVID symptoms?**		
No, I feel well	129	74.1
No, I do not need help	15	8.6
Yes, I want help	30	17.2

† , Two participants did not respond to this question.

Of the patients surveyed, 25.6% self-reported non-recovery from COVID-19 (‘feeling worse than before COVID-19’). Nearly 20% (30/174, 17.2%) stated that they wanted additional medical help for ongoing COVID-19 symptoms at two months post diagnosis. Of those who wanted help, 11 (37%) preferred assistance via a text message communication, six (20%) wanted a telephonic consultation, 13 (43%) desired a traditional clinic visit, and one (3%) wanted a community support group.

On multivariable analysis, neither sex, age, baseline severity of disease, or number of initial COVID-19 symptoms were predictive of self-reported dyspnoea, fatigue, ≥ 3 long COVID symptoms, or self-reported non-recovery. Self-reported non-recovery – or generally ‘feeling worse’ than prior to the SARS-CoV-2 infection – was highly predictive of having ≥ 3 long COVID symptoms in comparison to those participants who felt the same or better than prior to COVID-19 (OR: 14.99, 95% CI: 5.94, 37.84, *p* < 0.001).

## Discussion

The primary objective of this pilot study was to describe the prevalence of long COVID symptoms in patients with confirmed non-critical SARS-CoV-2 infection in Cape Town, South Africa two months after COVID-19 diagnosis. We found that 60% of patients who surveyed had at least one long COVID symptom, while 35% had ≥ 3 symptoms at two months following diagnosis. This is less than the NICD survey which found that 82% of patients 1-month post-hospitalisation had ≥ 1 persistent symptom and 66% of patients had ≥ 3 symptoms.^8^ However, the patients we surveyed had mild disease at baseline, whereas the NICD surveyed patients were hospitalised with COVID-19, so one would expect fewer persistent symptoms in the outpatient setting. Nonetheless, over a half of the patients had at least one long COVID symptom at two months, which is consistent with global findings that a significant number of patients with mild COVID-19 disease will experience ongoing morbidity.

The most common symptoms experienced by our patients were fatigue (35%), dyspnoea (20%), ageusia (20%), anosmia (18%), headaches (16%), and body aches (15%), all consistent with the spectrum of long COVID reported in both the literature and NICD study. The NICD patients had a higher rate of fatigue (70%), dyspnoea (32%), and headaches (18%), but that is expected with their more severe disease. These parallels suggest that South Africans with mild COVID-19 disease are at risk for a similar array of long COVID symptoms as their more severe counterparts, even if at a lower rate.

Although 60% of the patients who surveyed had ≥ 1 long COVID symptom and 35% had ≥ 3 symptoms at two months, only 25% of patients reported a feeling generally worse than prior to their COVID-19 infection. This mismatch between persistent COVID-19 symptoms and self-reported non-recovery suggests that, despite being surveyed on new or persistent symptoms, many patients may have experienced long COVID-like symptoms prior to infection. Given the high prevalence of poverty and chronic obstructive pulmonary disease (COPD) in Cape Town – over 20% of the population was estimated to have COPD^18^ – it is possible that many of the patients surveyed have undiagnosed lung disease and experienced fatigue and dyspnoea before and after their COVID-19 infection.^1^ Further study is needed to find out what proportion of these long COVID symptoms are directly related to the SARS-CoV-2 infection versus pre-existing morbidity.

Nonetheless, given the scale of mild COVID-19 infections in South Africa, even if 25% self-report non-recovery from their illness at two months post infection, that represents a significant number of people struggling with ongoing long COVID symptoms. In our study, 52% of employed patients missed work because of ongoing COVID-19 symptoms. About 24% of patients had additional clinical consultations for persistent symptoms outside of the isolation period. However, 71% of those visits were with private general practitioners. Only 7% of patients had follow-up consultations in the public sector, thus supporting our hypothesis that relatively few patients are utilising public primary healthcare facilities for ongoing long COVID treatment. During COVID-19 surges many primary healthcare services were de-escalated, or reduced, and rehabilitation professionals often redeployed to contact tracing teams.^19^ However, given that many patients cannot afford private healthcare, the public sector must make long COVID treatment and rehabilitation services more accessible at the primary healthcare level, even during ongoing COVID-19 waves.

Most patients who surveyed were very appreciative of the follow-up. One patient commented ‘I can’t believe the Department of Health care’s how I am doing’, a comment that suggests the public sector must be more patient-centred and welcoming to improve utilisation for non-urgent concerns. Nearly 20% of our patients wanted additional long COVID treatment, but over a half preferred remote consultations. Even if the public healthcare system does not have the capacity to offer in-person treatment and rehabilitation services for all its long COVID patients, our study suggests that many patients would be open to remote medical services that might be more feasible for the public sector to scale up for many long COVID patients.

Because of our small sample size, we were unable to identify baseline characteristics associated with developing long COVID. Larger studies are needed to identify which patients with mild COVID-19 disease are more likely to struggle with ongoing symptoms. However, we did find that patients with self-reported non-recovery were more likely to have ≥ 3 long COVID symptoms. This suggests that asking patients if they subjectively feel better might be a sufficient outpatient screen for identifying primary healthcare patients in need of long COVID treatment.

This study had several limitations. Firstly, because of the clinic population, we surveyed a racially homogenous sample. Although we have no reason to believe that South Africans of different racial backgrounds would have different disease symptoms, they might have different health-seeking behaviours or access to care. Secondly, because of testing restrictions in the public sector, our study population was older than the general population, which could bias results given that younger patients are more likely to have mild or asymptomatic disease. Thirdly, we did not survey the psychological impact of COVID-19 in the primary healthcare setting, information that is also needed to shape the primary healthcare response to long COVID. Fourthly, we chose to survey patients at two months post diagnosis to compare to prior studies. Further study is needed to identify the proportion of patients experiencing long COVID at one month, three months and beyond to understand the full extent of morbidity.

Fifthly, because of study resource constraints, we surveyed a relatively small sample of patients. Additional studies are needed on a population level to confirm if our results are generalisable to the broader South African population. Nonetheless, given that no other data exists documenting the extent of long COVID in mild COVID-19 patients in South Africa, we believe that this study provides a valuable start to documenting the prevalence of long COVID in COVID-19 patients who were not hospitalised.

## Conclusion

We found a high prevalence of persistent long COVID symptoms at two months in patients with mild COVID-19 illness, but poor utilisation of the public sector for treatment. The public sector must provide primary healthcare treatment for long COVID patients who otherwise might suffer ongoing morbidity because of their symptoms.
